# Fabrication and Evaluation of N-Channel GaN Metal–Oxide–Semiconductor Field-Effect Transistors Based on Regrown and Implantation Methods

**DOI:** 10.3390/ma13040899

**Published:** 2020-02-18

**Authors:** Huu Trung Nguyen, Hisashi Yamada, Toshikazu Yamada, Tokio Takahashi, Mitsuaki Shimizu

**Affiliations:** GaN Advanced Device Open Innovation Laboratory (GaN-OIL), National Institute of Industrial Science and Technology (AIST), Nagoya 4648601, Japan; hisashi-yamada@aist.go.jp (H.Y.); toshi.yamada@aist.go.jp (T.Y.); tokio-takahashi@aist.go.jp (T.T.); mitsu.shimizu@aist.go.jp (M.S.)

**Keywords:** gallium nitride, metal-oxide field-effect transistors, fabrication

## Abstract

We have demonstrated the enhancement-mode n-channel gallium nitride (GaN) metal-oxide field-effect transistors (MOSFETs) on homoepitaxial GaN substrates using the selective area regrowth and ion implantation techniques. Both types of MOSFETs perform normally off operations. The GaN-MOSFETs fabricated using the regrowth method perform superior characteristics over the other relative devices fabricated using the ion implantation technique. The electron mobility of 100 cm^2^/V·s, subthreshold of 500 mV/dec, and transconductance of 14 μs/mm are measured in GaN-MOSFETs based on the implantation technique. Meanwhile, the GaN-MOSFETs fabricated using the regrowth method perform the electron mobility, transconductance, and subthreshold of 120 cm^2^/V s, 18 μs/mm, and 300 mV/dec, respectively. Additionally, the MOSFETs with the regrown p-GaN gate body show the I_on_/I_off_ ratio of approximately 4 × 10^7^_,_ which is, to our knowledge, among the best results of GaN-MOSFETs to date. This research contributes a valuable information for the design and fabrication of power switching devices based on GaN.

## 1. Introduction

Gallium nitride (GaN) possesses such extraordinary characteristics as high breakdown electric field (3 MV/cm) [1], high saturation velocity (1.4 × 10^7^ cm/s) [1], high electron mobility, and good thermal conductivity (~100 W/m·K) [1], thus emerging as one of the most promising materials for next generation power switching devices. Among GaN-based transistor structures, heterostructure field-effect transistors (HFETs) based on AlGaN/GaN heterojunction have been widely used for high-power and high-frequency applications owing to the high electron mobility deriving from the two-dimensional electron gas (2DEG) source. However, because HFETs operate at the normally on mode, there are many significant disadvantages, such as large leakage current, high power consumption in their analog and/or power applications. In order to demonstrate normally off GaN-based HEMTs, several approaches have been proposed. In general, a thin AlGaN barrier layer with a low Al concentration is usually used to achieve the 2DEG depletion region in normally off GaN-based HEMTs. Because the depletion of the 2DEG channel must be formed under the gate, the recession structure of the AlGaN barrier layer has been considered as one of effective solutions for normally off GaN HEMTs [2]. However, an accurate control of the AlGaN etching technology is highly required in this method. Moreover, the damages derived from the etching process probably increase the gate leakage current and hysteresis of threshold voltages. Recently, p-GaN (or p-AlGaN) layer on the AlGaN/GaN heterostructure has been used as a promising approach to demonstrate normally off GaN-based HEMTs [3,4]. However, this structure is more complicated than previous candidates. 

To solve this issue, approaches to use GaN-based metal/oxide/semiconductor field-effect transistors (MOSFETs) have been usually proposed by many researchers [5,6,7,8,9,10,11,12,13]. GaN-MOSFET is an ideal candidate to overcome the weaknesses of GaN-HFET owing to its large voltage sweep range, low gate leakage currents, and structure simplicity. Additionally, an enhancement-mode operation of n-channel GaN-MOSFETs is crucial in avoiding the turn-off failure for the circuit safety of the integrated circuit (IC) applications. Not only the threshold voltage control so that the GaN-based MOSFETs perform the normally off characteristic, but also the low on-resistance is very important for transistors designed for power switching applications. The on-resistance is significantly affected by the channel electron mobility. A mobility of beyond 100 cm^2^/ V·s is considered as a standard to obtain the on-resistance of 1 mΩ·cm^2^, which is required for recent power IC systems [9]. 

The quality of GaN films, treatment of gate dielectrics, structural optimization, and relative fabrication processes are among crucial issues to optimize the n-channel GaN-MOSFETs with the high electron channel mobility, low interface state density, low gate leakage current and large I_on_/I_off_ properties [11,12,13,14,15,16,17,18,19,20,21]. One of the most essential points is the formation of the high-quality GaN-channels (S/G/D regions). Recently, implantation and regrowth are the most common methods to produce high-quality n- and p-doped GaN. Many researchers have solely evaluated the quality of n- and p-GaN layers produced by the implantation and epitaxial growth [22,23]. However, diverse applications of these methods to fabricate actual GaN-MOSFETs have not been fully explored yet. For instance, the advantages and disadvantages of GaN-MOSFETs fabricated using both methods still remain unclear. Another issue of fabricating high-performance n-channel GaN-MOSFETs is the interface state density between dielectrics and semiconductors. Various dielectrics, such as SiO_2_, Al_2_O_3_, SiN_x_ AlSiO_x_, HfO_2_, etc., have been widely used in general MOSFETs. Among them, Al_2_O_3_ is considered as an excellent material owning to the high dielectric constant (8–10), good breakdown field (>1 MV/cm), and large bandgap (6–8 eV). Because of the capability to synthesize precisely the thickness and pinhole-free thin film with accurate monolayers, the atomic layer deposition (ALD) is usually used to deposit Al_2_O_3_ as a gate dielectric in MOSFETs. In general, a pre-treatment is very important to clean the top poor-quality GaN layer with the native oxidation and impurities that cause high-density interface states (>10^12^ cm−^2^eV^−1^) because of the insufficient chemical bonds during the forming process of Al_2_O_3_. Many attempts have been done to prepare good GaN surfaces for ALD-Al_2_O_3_ deposition [24,25,26,27]. Usually, acid cleaning is used to remove the native oxidized layer and impurities in GaN-capacitors. However, during the complicated fabrication process of GaN-MOSFETs prior to the deposition of dielectric layers, p-GaN channels contain significant damages and defects, thus resulting in higher surface state density than that of general GaN-capacitors. This indicates an essential necessity for an alternative surface treatment processing for GaN-MOSFETs fabrication. 

To solve the above issues, we fabricated n-channel GaN-MOSFETs by both implantation and regrowth methods. For the first time, the inversion-mode GaN-MOSFETs are fabricated with regrown p-GaN layers after forming the epitaxially grown n-GaN layers. In this method, it is expected to minimize the damages on the vulnerable p-GaN layer during the complicated fabrication process. The traditional implantation technique is also used to fabricate n-channel GaN-MOSFETs and compared with the proposed regrowth method. Additionally, the semiconductor surface treatments using the combination of physical and chemical treatments prior to the dielectric deposition have been conducted to reduce the trapped charge density. The ozone oxidation technique is used to form the sacrificial GaO_x_ on the GaN film, which is subsequently removed by wet etchings. Therefore, the damaged GaN layer consisting of undesired defects during the fabrication process would be partly avoidable. As a result, the MOSFETs with the regrown p-GaN gate body show the superior properties over the other devices fabricated using the implantation method. According to our knowledge, the on/off ratio (I_on_/I_off_) of approximately 4 × 10^7^ obtained in this work is one of the best results to date.

## 2. Experimental

**Figure 1:**
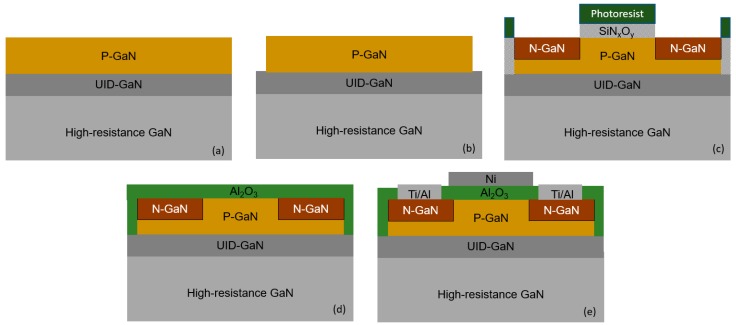
Fabrication process of the gallium nitride-metal-oxide field-effect transistors (GaN-MOSFETs) fabricated using the implantation technique. (**a**) p-GaN grown on a free standing GaN substrate, (**b**) mesa structure, (**c**) implantation of Si atoms, (**d**) Al_2_O_3_ deposition, (**e**) contact deposition and annealing.

**Figure 2:**
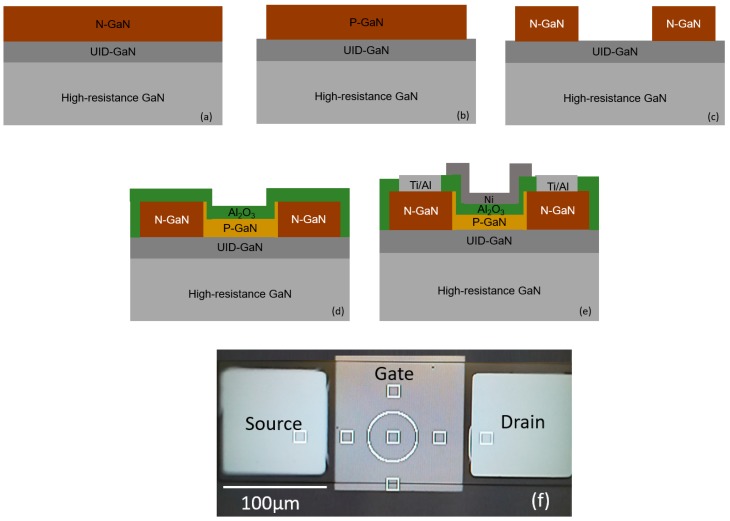
Fabrication process of the gallium nitride-metal-oxide field-effect transistors (GaN-MOSFETs) fabricated using the regrowth method. (**a**) n-GaN grown on a free standing GaN substrate, (**b**) mesa structure, (**c**) etching n-GaN layer on the gate-body region, (**d**) regrowth of p-GaN and Al_2_O_3_ deposition, (**e**) contact deposition and annealing, (**f**) the top view of the fabricated MOSFETs.

The fabrication of the GaN-MOSFET structure with the implantation technique starts with the 500 nm-thick homoepitaxial Mg-doped p-GaN (2 × 10^17^ cm^−3^) layer grown by MOCVD on a free standing GaN substrate consisting of a thin unintentionally doped GaN (UID- GaN) and high-resistance insulation GaN substrate, as shown in Figure 1a. The SiO_2_ hard mask is deposited on the top of the free standing GaN structure using the plasma enhanced *chemical vapor deposition* (PECVD) with tetraethyl orthosilicate and oxygen. Subsequently, mesa structures are defined using the conventional lithography and wet etching of the SiO_2_ hard mask. The GaN mesa-insulation regions are formed using Cl_2_/BCl_3_ reactive-ion etching, as shown in Figure 1b. The SiO_2_ hard mask is removed using the buffered hydrofluoric (BHF) acid. Subsequently, the source and drain (S/D) regions are implanted with Si atoms at room temperature with 10 nm-thick SiO_x_N_y_ and photoresist protection layer on the gate regions, as shown in Figure 1c. The implantation energy is 15 keV and the dose implantation is 10^15^ cm^−2^. The rapid thermal annealing (RTA) in the N_2_ atmosphere at 1000 °C is used to activate the dopants after the implantation. For the GaN-MOSFET structure fabricated by the regrowth method, the fabrication process is based on the n-GaN (3 × 10^18^ cm^−3^), as shown in Figure 2a. The GaN mesa-insulation regions and n-GaN layer on the gate-body region are etched using the reactive-ion etching with Cl_2_/BCl_3_ gas mixture, as shown in Figure 2b,c. The sample is immerged in the tetramethyl ammonium hydroxide (TMAH) with 10 wt% at 70 °C in 5 min to etch the damaged n-GaN on the vertical side because of the reactive-ion etching. Subsequently, 200 nm-thick Mg-doped p-GaN (2 × 10^17^ cm^−3^) is selectively regrown on the gate region. The regrown p-GaN layer is formed using trimethylgallium (TMGa), bis (cyclopentadienyl) magnesium (Cp_2_Mg), and NH_3_ as precursors at 950 °C. The growth is performed with the NH_3_ flux of 10 slm and TMGa flux of 20 sccm. An ozone oxidation at 300 °C is used to form the sacrificial GaO_x_ of the damaged GaN layer, which is subsequently removed using BHF acid. The treatment of forming and removing the sacrificial GaO_x_ layer is repeated two times and re-cleaned using diluted hydrofluoric (DHF) acid and HCl acid with the concentration of 0.8 M (mole/litter). The 28 nm-thick Al_2_O_3_ thin film as a dielectric is deposited using PEALD with trimethylaluminum and oxygen plasma, as shown in Figure 1d and Figure 2d. According to previous studies, the Al_2_O_3_ thin film transforms from amorphous to crystalline phase at over 800 °C [28]. Therefore, Al_2_O_3_ dielectric is annealed at 700 °C in a nitrogen (N_2_) ambience for 1 minute using the rapid temperature annealing (RTA) technique. The electron beam evaporation and conventional wet etching methods are used to form the Ni gate electrodes. The Ti/Al stack structure is formed on the S/D regions by the lift-off technique and subsequently treated with the metallization annealing (PMA) at 600 °C in a N_2_ ambience to obtain the ohmic contact, as shown in Figure 1e and Figure 2e. The top view of the fabricated MOSFETs is shown in Figure 2f.

## 3. Results and Discussion

According to the previous studies, interface state densities (*D_it_*) of approximately 10^12^ cm^−2^eV^−1^ and higher are observed in the GaN-MOS capacitors without the surface treatment [29,30]. Because GaN cannot be formed with an oxides-free pristine interface, pre-deposition surface treatments are crucial for a good nucleation of dielectric layers. An erratic nucleation can lead to the formation of such defects as vacancies, vacancy-complexes, interstitials, etc., resulting in high densities of interface traps and fixed charges. By using the proposed treatment process, a low *D_it_* can be obtained because of the removal of nitrogen vacancy (*N_v_*), Ga dangling bonds, and damages on the surface of GaN layer. As a result, the surface trapped state density of approximately 10^11^ cm^−2^eV^−1^ is obtained in our previous work [31]. This value is low enough to satisfy the operation of general MOSFETs. 

**Figure 3:**
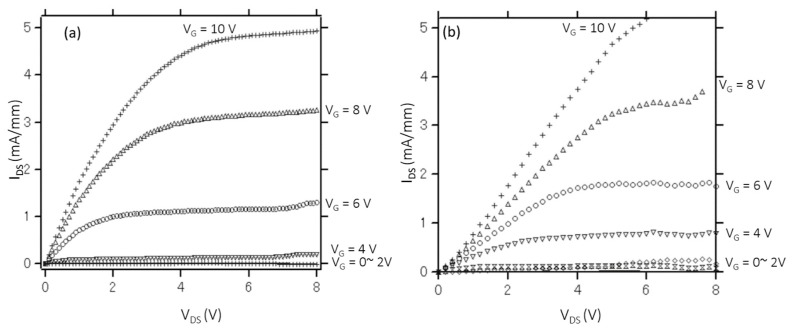
Output current–voltage (I_d_–V_d_) characteristics of GaN-MOSFETs fabricated using the implantation technique (**a**) and regrowth method (**b**).

Two types of GaN-MOSFETs fabricated with different techniques are evaluated and compared. Figure 3 shows the output current–voltage (I_d_–V_d_) characteristics of the GaN- MOSFETs with the channel length and channel width of 100 µm. It has been observed that the drain currents are effectively controlled by the gate voltages, thus confirming the transistor operation. Both devices operate with the normally off (enhancement-mode) function. Additionally, the maximum saturation source-drain current of the GaN-MOSFET fabricated using the regrowth method is higher than that of the implantation technique, revealing the low on-state resistance and high electron mobility.

**Figure 4:**
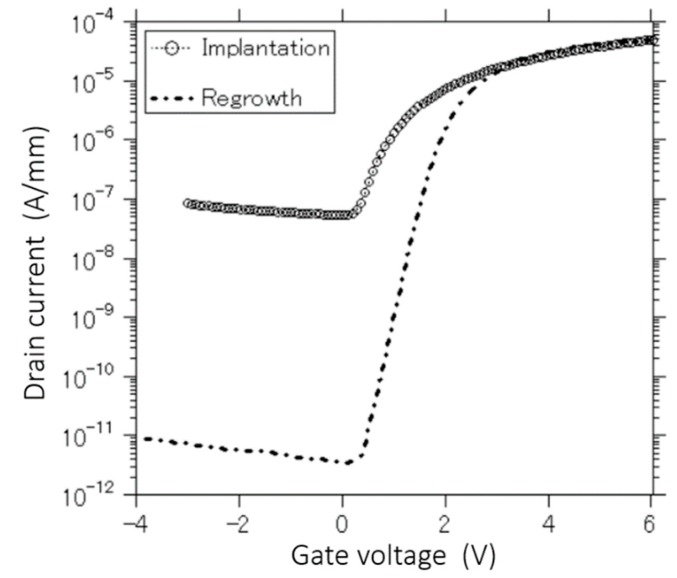
Transfer I_d_–V_g_ characteristics.

The logarithmic plots of the drain current – gate voltage (I_d_–V_g_) transfer characteristics under the drain-to-source voltage of 50 mV at a room temperature are shown in Figure 4. The threshold voltages of the devices fabricated by the regrowth and ion implantation methods extracted from the gate bias intercept of the linear extrapolation I_d_ are approximately 1.6 and 2.0 V, respectively. Additionally, the leakage current below the pinch-off voltage in the MOSFET fabricated using the regrowth method is roughly 4 orders of magnitude better than that of the ion implantation technique. This behavior might be explained as the influence of damages after the ion implantation process. During the implantation process, it is difficult to control defect states although the post deposition annealing (PDA) process can partly release this undesired behavior. According to the previous discussions, there is a possibility that defect states play a role of donors in p-GaN and thus dominating the off-state currents. The high leakage current might be caused by the defects, dislocation, and trapped electrons that create the sub-threshold channel in the off-region. Additionally, the subthreshold slope of device fabricated using the regrowth method is approximately 300 mV/dec, which is better than that of the device fabricated by the ion implantation technique (~ 500 mV/dec). This difference is also related to the drain leakage current of the implanted device as a previous discussion. It can be concluded that it is possible to reduce the damages on p-GaN layer during the fabrication process by using the fabrication process based on the regrowth method. Therefore, the off-state current is much lower than that of the device fabricated by the implantation technique. The performances of regrown devices in this work are better than the previous inversion-channel GaN MOSFETs using MgO and SiN_x_ and other Al_2_O_3_ dielectrics [5,18,32,33]. According to our knowledge, the I_on_/I_off_ ratio of approximately 4 × 10^7^ obtained in this work is one of the best results to date.

**Figure 5:**
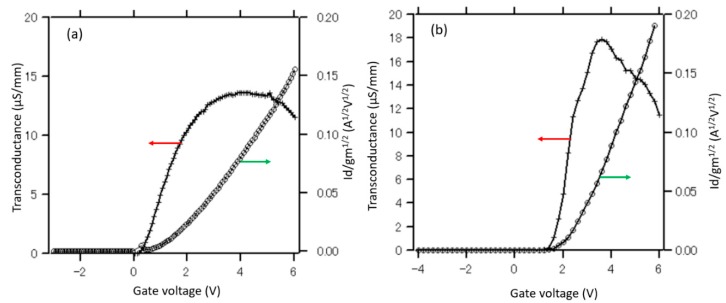
Y-function and transconductance of MOSFETs fabricated using the ion implantation technique (**a**) and regrowth method (**b**).

**Figure 6:**
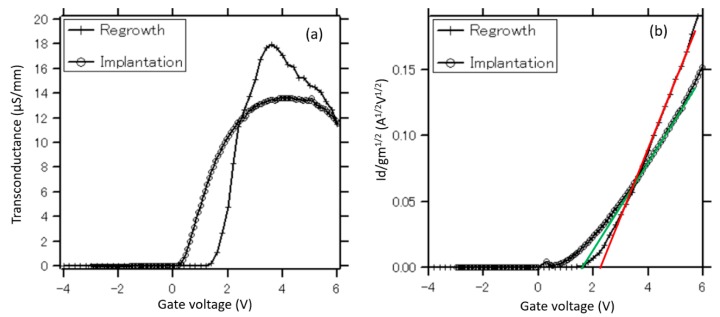
Comparison of transconductances **(a)** and threshold voltages **(b)** extracted from Y-function of two types of MOSFETs.

**Figure 7:**
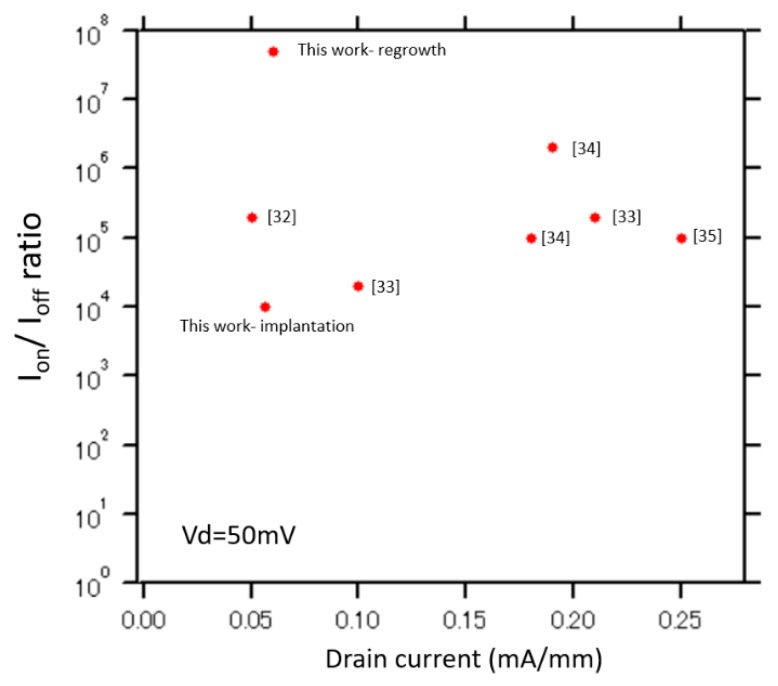
Comparison of I_on_/I_off_ ratios obtained in this work and previous reports.

Figure 5a,b shows the plots for the Y-function and transconductance of the MOSFETs fabricated using the implantation technique and regrowth method, respectively. The comparisons of the transconductance and Y-function of two devices are shown in Figure 6a,b, respectively. According to Figure 6a, the transconductance of the device fabricated by the regrowth method is larger than that of the implantation technique. Moreover, the threshold voltages of the implanted GaN-MOSFET and regrown GaN-MOSFET extracted from the Y-function are approximately 1.8 and 2.2 V, respectively, indicating a good p/n-GaN junction in the device fabricated by the regrowth method. These values are slightly higher than those extracted from the gate-bias intercept of the linear extrapolation of I_d_ in the I_d_–V_g_ characteristic. The minor difference of threshold voltages between two extraction methods is considered to be due to the fitting error. In general, the selectively etched and regrown p-GaN channel is expected to enhance the drain current, channel electron mobility, and reduce the series resistance by reducing the damages on p-GaN channels. Although the gate channel of the implanted MOSFET is fully covered by SiN_x_O_y_ and photoresist during the Si-implantation process, it is suggested that some Si atoms still penetrate the p-GaN layer. Moreover, during the fabrication process, the p-GaN layer is exposed in the plasma environment, high temperature activation annealing (~ 1000 °C), resulting in many defects in the structure. Therefore, the channels of MOSFET fabricated using the regrowth method are expected to possess more extensive p/n junction than those of the MOSFET fabricated using the implantation technique. In contrast, because of the defects, electrons diffusing and/or contaminating in the p-GaN layer of the device fabricated by the implantation technique, the devices investigated in this work do not only utilize the channel inversion as usual inversion- mode MOSFETs but also the electron accumulation formed at the surface of the damaged p-GaN layer. Since there are defects and electrons trapped in the structure, the drain current is not only controlled by adjusting the gate bias and interface conditions, but also the amount of the subthreshold carriers. From above discussions, it is shown that the GaN-MOSFETs fabricated using the regrowth method perform superior characteristics of threshold voltage, on-state current, transconductance, and subthreshold behaviors over the other relative devices fabricated using the ion implantation technique. The comparison of I_on_/I_off_ ratios obtained in this work and previous reports is shown in Figure 7 [32,33,34,35]. It is concluded that the I_on_/I_off_ ratio of GaN-MOSFETs can be optimized using the regrowth and surface treatment techniques proposed in this work.

**Figure 8:**
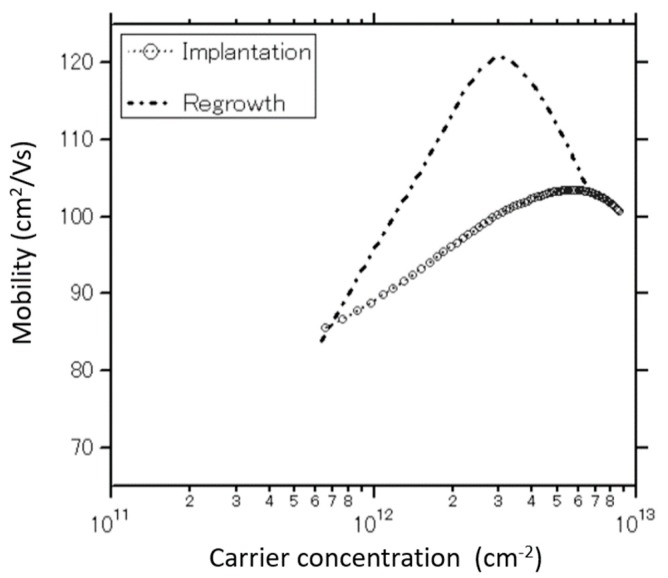
Electron mobilities in MOSFETs fabricated using the regrowth and implantation method.

Figure 8 shows the effective channel-electron mobility of fabricated GaN-MOSFETs. The effective electron mobilities are extracted from the Id–Vg characteristics measured in the dark room. The electron mobilities obtain the maximum values at the specific applied bias gate voltages before decreasing with the increase of carriers density because of the increase of the applied gate voltages. This is the evidence of the appearance of the Coulomb scattering centers. Additionally, the maximum mobility of 120 cm^2^/V·s in the regrown MOSFET is higher than that (100 cm^2^/V·s) of the implanted MOSFET. Because of the damages caused by the implantation and other fabrication steps, the p-GaN layer can contain defects, trapped electrons, ionized impurities, etc. Impurity scattering derived from the charged particles caused by those undesired behaviors would make the electron mobility in the channel formed at the GaN-surface considerably lower than that of a normal bulk GaN. Matthiessen’s rule has shown many factors deciding the channel mobility, such as Coulomb scattering mobility, surface phonon mobility, bulk mobility, and surface roughness mobility. Regarding the influence of interface charges near the MOS interface on the channel electron mobility, a significant mobility degradation is observed in Si-MOSFETs with the interface state density of over 10^12^ cm^−2^eV^−1^ [36]. According to the Terman method, the interface state density for the fabricated devices is approximately 10^11^ cm^−2^eV^−1^. Therefore, it is expected that the mobility of the MOS channel region will be comparable to the bulk GaN’s mobility. A bulk mobility of approximately 600 cm^2^/V·s at room temperature has been reported for n-doped bulk GaN with N_d_ = 3 × 10^17^ cm^−3^ [37]. However, although the interface trapped density obtained in this work is low enough for general MOSFETs, the maximum inversion electron mobility is five times lower than that of the bulk GaN. Therefore, it seems that the low channel mobility in GaN-MOSFETs is mainly attributed by GaN crystallinity and/or coexistence of carriers originated from defect states rather than the oxide and/or interface trapped states. It is expected that higher hole mobilities can be achieved by further improvement of the MOSFETs processing and p-GaN quality.

## 4. Conclusions

In this work, we have fabricated and evaluated many MOSFET devices based on homoepitaxial GaN using two methods of the selective area regrowth and ion implantation. For the implantation method, the source and drain regions are implanted with Si atoms with 10 nm-thick SiO_x_N_y_ and photoresist protection layer on the gate regions. For the regrown method, the gate is completely removed and regrown after making the source and drain regions to minimize damages on the gate. Moreover, the semiconductor surface treatments using the combination of physical and chemical treatments prior to the dielectric deposition have been proposed for the GaN-MOSFET’s fabrication to reduce the trapped charge density. As a result, all characteristics of MOSFETs fabricated using the regrowth method are better than those of the relative devices fabricated using the ion implantation technique. The electron mobility, transconductance, and subthreshold values in GaN-MOSFETs based on implantation technique are 100 cm^2^/V·s, 14 μs/mm, 500 mV/dec, respectively. Meanwhile, the GaN-MOSFETs fabricated using the regrowth method perform the electron mobility, transconductance, and subthreshold of 120 cm^2^/V·s, 18 μs/mm, 300 mV/dec, respectively. The MOSFETs with the regrown p-GaN gate body show the I_on_/I_off_ ratio of approximately 4 × 10^7^_,_ which is, to our knowledge, among the best results of GaN-MOSFETs to date. This investigation provides a valuable information for the future work on GaN-MOSFETs for applications of high-power switching devices.
